# Duration of constant rate infusion with diazepam or propofol for canine cluster seizures and status epilepticus

**DOI:** 10.3389/fvets.2023.1247100

**Published:** 2023-08-22

**Authors:** Giulia Cagnotti, Sara Ferrini, Giorgia Di Muro, Eleonora Avilii, Alessandra Favole, Antonio D’Angelo

**Affiliations:** ^1^Department of Veterinary Sciences, University of Turin, Turin, Italy; ^2^Istituto Zooprofilattico del Piemonte, Liguria e Valle d’Aosta, Turin, Italy

**Keywords:** diazepam, dog, epilepsy, propofol, status epilepticus

## Abstract

**Introduction:**

Constant rate infusion (CRI) of benzodiazepines or propofol (PPF) is a therapeutic option for cluster seizures (CS) and status epilepticus (SE) in canine patients non-responding to first-line benzodiazepines or non-anesthetics. However, specific indications for optimal duration of CRI are lacking. The aim of this study was to determine the effect of duration of anesthetic CRI on outcome and length of hospital stay in dogs with refractory seizure activity of different etiology.

**Study design:**

Open-label non-randomized clinical trial.

**Materials and methods:**

Seventy-three client-owned dogs were enrolled. Two groups [experimental (EXP) vs. control (CTRL)] were compared. The EXP group received diazepam (DZP) or PPF CRI for 12 h (±1 h) and the CTRL group received DZP or PPF CRI for 24 h (±1 h) in addition to a standardized emergency treatment protocol identical for both study groups. The historical control group was made up of a population of dogs already reported in a previously published paper by the same authors. Favorable outcome was defined as seizure cessation after CRI, no seizure recurrence, and clinical recovery. Poor outcome was defined as seizure recurrence, death in hospital or no return to acceptable clinical baseline. Univariate statistical analysis was performed.

**Results:**

The study sample was 73 dogs: 45 (62%) received DZP CRI and 28 (38%) received PPF CRI. The EXP group was 39 dogs (25 DZP CRI and 14 PPF CRI) and the CTRL group 34 dogs (20 DZP CRI and 14 PPF CRI). We found no statistically significant difference in outcomes between the groups. The median length of stay was 56 h (IQR, 40–78) for the ALL EXP group and 58.5 h (IQR, 48–74.5) for the ALL CTRL group (*p* = 0.8).

**Conclusion:**

Even though a shorter DZP or PPF CRI duration was not associated with a worse outcome, the study failed to identify a clear superiority of shorter CRI duration on outcome or length of hospital stay in dogs with refractory seizure activity of different etiology.

## 1. Introduction

Constant rate infusion (CRI) of benzodiazepines or propofol (PPF) is an option for treating cluster seizures (CS) or status epilepticus (SE) in canine patients non-responding to first-line benzodiazepines and non-anesthetics (1–3). While the use of AED and anesthetic CRI is part of the therapeutic protocol for refractory seizure activity in many veterinary clinics and hospitals (4–6), specific indications for optimal CRI duration are lacking, however.

Induction of therapeutic coma (24–48 h) is recommended in human patients with super-refractory status epilepticus (7, 8). While administration of CRI has been associated with increased risk of mortality, complications, and prolonged hospital stay (9–13), recent observational data suggest a higher risk of mortality, complications, and longer hospital stay associated with protracted CRI (14).

Only two retrospective studies published to date describe the use of benzodiazepines or PPF CRI in dogs suffering CS and SE of different etiology, but neither study evaluated the effect of CRI duration on seizure control and outcome (1, 2). In particular one of the two mentioned retrospective studies has been performed by the same authors of this study (2). After collecting consistent data on the retrospective population of dogs administered with diazepam (DZP) or PPF CRI, we decided to exploit this information to investigate further the utilization of these treatment protocols based on the recent suggestions from studies performed in human medicine.

The median CRI duration in the aformentioned studies was 25 and 24 h, respectively (1, 2). To the authors knowledge, very scant information on minimum CRI duration is available from the literature. According to a recent veterinary review, infusions in case of SE should be continued for at least 6 h (4), while elsewhere it is reported that the CRI dosage rate should be reduced by 50% every 6 h for at least two reductions before discontinuing the drug (6). By combining these recommendations, a 6 to 12 h CRI seems to be the minimum duration potentially justified.

With these observations in mind, we conducted this prospective study to compare the effect of different anesthetic CRI duration in canine epileptic patients with refractory seizure activity presented to a single veterinary teaching hospital. Our hypothesis was that shorter CRI would be as efficacious as longer duration for controlling seizure activity and might also shorten length of hospital stay.

## 2. Materials and methods

### 2.1. Study design

The study was approved by the Bioethics Committee of the University of Turin (approval number: 0598251). Written informed consent was obtained from the dogs’ owners before enrollment.

For this non-randomized clinical trial, we compared two groups [experimental (EXP) group vs. control (CTRL) group] to evaluate the effect of CRI duration with DZP or PPF on clinical outcome and length of hospital stay in canine patients suffering refractory seizure activity. The EXP group dogs were enrolled prospectively from October 2021 to February 2023 and received CRI for 12 h (±1 h); the CTRL group received CRI for 24 h (±1 h) and included dogs from a previous study (2). CRI was administered in addition to standardized emergency treatment and was identical in both study groups (see Treatment Protocol).

### 2.2. Study population and definitions

#### 2.2.1. EXP group

Dogs presented to the Veterinary Teaching Hospital of the Department of Veterinary Sciences of Turin, between October 2021 and February 2023 for epileptic CS or SE of any etiology were eligible for inclusion in the study. No limitations on age, breed or sex were applied.

CS were clinically defined as the occurrence of two or more epileptic seizures within a 24-h period; SE was defined as convulsive seizure activity lasting more than 5 min or the occurrence of two or more epileptic seizures without complete recovery of consciousness in between (15).

Inclusion criterion was the requirement of a DZP or PPF CRI for the control of epileptic seizure activity refractory to a standardized emergency treatment protocol in place at our institution (see Treatment Protocol). Dogs were excluded if they had received emergency treatment other than by protocol according to standardized guidelines, the medical chart data on CRI were incomplete, and if the patient had not been directly supervised by a board-certified neurologist or a neurology resident under the supervision of a board-certified neurologist. Only the data from the first of multiple hospitalizations during the study period were analyzed for the present study.

Seizure etiology was classified according to the International Veterinary Epilepsy Task Force (15). Reactive seizures were defined as a history of possible or confirmed exposure to toxic agents or based on blood test results. A diagnosis of idiopathic epilepsy (IE) was made as follows: if the first seizure occurred between 6 months and 6 years of age; if the interictal neurological examination was normal [except for antiepileptic drug (AED)-induced neurologic abnormalities and post-ictal neurologic deficits]; if no clinically significant abnormalities were identified on minimum database blood tests comprising electrolytes, plasma ammonia concentration, and bile acid stimulation test (tier I confidence level); if the findings from brain magnetic resonance imaging (MRI) and cerebrospinal fluid (CSF) analysis were unremarkable (tier II confidence level). Structural epilepsy was diagnosed when reactive causes of seizures were ruled out, along with a compatible signalment, history, abnormal interictal neurological examination (suspected structural epilepsy), and anomalous MRI and CSF findings or structural causes confirmed at necropsy (confirmed structural epilepsy). Dogs were classified as having undefined epilepsy if none of the investigations could be performed and no follow-up data were available for proper classification.

#### 2.2.2. CTRL group

The historical control group was made up of a population of dogs already reported in a previously published paper by the same authors (2). This population included dogs that suffered CS or SE and received CRI treatment (September 2016–December 2019) at our institution, according to the same inclusion and exclusion criteria in place for the EXP group of the present study, and were etiologically classified by the same principles. Only canine patients that received CRI for 24 h (±1 h) were included. Only the data from the first of multiple hospitalizations were analyzed.

### 2.3. Treatment protocol

The standardized emergency treatment protocol was identical for both study groups and entailed rectal/IV administration of DZP (1–2 mg/kg) if the dog was actively seizuring at presentation, followed by IV phenobarbital (PB) (4–5 mg/kg q8h) and rectal levetiracetam (LEV) (40 mg/kg one shot). CRI of DZP (0.5 mg/kg/h) or PPF (0.1–0.2 mg/kg/min) was initiated if seizure activity persisted or recurred despite emergency treatment. PPF CRI was considered when seizure activity did not resolve with emergency treatment. DZP CRI was initiated if convulsive activity resolved initially with emergency treatment protocol but then recurred within a few hours or if convulsive activity persisted after initial improvement.

### 2.4. Outcome measures

#### 2.4.1. Outcome after CRI

Favorable outcome was defined as cessation of clinically visible seizure activity within a few minutes after initiation of CRI, no seizures recurred within the first 24 h after discontinuation of CRI through to hospital discharge, and good clinical recovery. Poor outcome was defined as recurrence of seizure activity despite treatment or death in hospital (either by euthanasia or spontaneous) because of recurrent seizures, catastrophic consequences of prolonged seizures (e.g., cardiac arrhythmias, acute renal failure, rhabdomyolysis, hemorrhagic diarrhea, ab ingestis pneumonia) or no return to an acceptable neurological and clinical baseline, despite apparent control of seizure activity.

#### 2.4.2. Length of hospital stay

Length of hospital stay was defined as the time between admission and discharge expressed in hours and was recorded for dogs that survived and were discharged alive.

### 2.5. Statistical analysis

Descriptive statistics and statistical analyses were performed using commercially available software (R version 4.1.3—November 2021). Continuous variables were tested for normality distribution using the Shapiro-Wilk test and found to not normally distributed. Standard descriptive statistics are reported as median and interquartile range (IQR) for continuous variables and as percentage and frequency for categorical variables.

Three scenarios were investigated. The first considered all cases receiving CRI for 12 h (ALL EXP group) versus those receiving CRI for 24 h (ALL CTRL group), regardless whether with DZP or PPF. Further evaluations were then performed based on drug and CRI duration: DZP EXP group (DZP CRI for 12 h) versus DZP CTRL group (DZP CRI for 24 h) and PPF EXP group (PPF CRI for 12 h) versus PPF CTRL group (PPF CRI for 24 h).

Homogeneity between the two groups in the three scenarios was studied with the chi-square test, Fisher’s two-tailed exact test, and Wilcoxon ranked-sum two-tailed test. Suspected and confirmed IE, suspected and confirmed structural epilepsy, and all purebred dogs were grouped together for statistical comparison.

Comparison between the number of dogs with favorable outcome and those with poor outcome in relation to CRI duration in the three scenarios was carried out using the chi-square test or Fisher’s two-tailed exact test as appropriate. Comparison between length of hospital stay and CRI duration in the three scenarios was carried out using Wilcoxon ranked-sum two-tailed test.

The same statistical analyses were then performed focusing only on the population of dogs affected by IE.

Statistical significance was set at *p* < 0.05.

## 3. Results

A total of 125 dogs receiving CRI for CS or SE at our institution during the study period were recorded. Fifty-two cases were excluded because of previous hospitalization with CRI administration (*n* = 20), because of non-standardized CRI duration (*n* = 12), because of incomplete medical records (*n* = 9), because of non-standardized emergency treatment (*n* = 6), and because management of convulsive activity was not directly supervised by a board-certified neurologist or a neurology resident (n = 5). The present study population was therefore 73 dogs. Of these, 39/73 dogs were prospectively enrolled in the EXP group during the study period, while 34/73 patients were included as historical CTRL group from a previous study performed by the same authors (2).

Mixed breed was the most numerous (26/73, 36%), followed by Border Collie (6/73, 8%), and Corso Dog (5/73, 7%). Forty-three out of 73 (59%) dogs were male (40/43 intact, 3/43 neutered) and 30/73 (41%) were female (14/30 intact and 16/30 neutered). Median weight was 23 kg (IQR, 14.5–33) and median age at inclusion was 61.5 months (IQR, 34–96). CS was diagnosed in 45/73 (62%) and SE in 28/73 dogs (38%). IE was diagnosed in 36/73 (49%) dogs (15/36 tier I and 21/36 tier II) and structural epilepsy in 16/73 (22%) dogs (7/16 suspected and 9/16 confirmed). A cause of reactive seizures was identified in 14/73 (19%) dogs, whereas no reason for seizure activity could be identified in 7/73 (10%) dogs, which were categorized as having undefined epilepsy. Most dogs had a history of seizures (50/73, 68%), 40/50 (80%) of which were receiving antiseizure medication (AED): 24/40 (60%) were receiving 1 AED; 11/40 (28%) were receiving 2 AEDs, 4/40 (10%) were receiving 3 AEDs, and 1/40 (2%) was receiving 4 medications.

A total of 45/73 (62%) dogs received DZP CRI and 28/73 (38%) PPF CRI. The characteristics of the two main study groups (ALL EXP group and ALL CTRL group) are presented in Table 1. There was no statistically significant difference between the two groups. Further evaluation of homogeneity between the groups receiving DZP CRI (DZP EXP group and DZP CTRL group) and PPF CRI (PPF EXP group and PPF CTRL group) showed no statistically significant differences (Tables 2, 3).

**Table 1:** Baseline characteristics of the study groups.

Characteristic		ALL EXP group (*n* = 39)	ALL CTRL group (*n* = 34)	*p* value
Breed	Cross breed	15	11	
	Pure breed	24	23	0.6
Sex	Female intact	7	7	
	Female neutered	12	4	
	Male intact	18	22	
	Male neutered	2	1	0.2
Weight (kg)		23 (9.75–34.5)	23 (16.10–29.18)	0.9
Age at inclusion (months)		66 (34–111)	54 (22–90.75)	0.2
Seizure etiology	Idiopathic	18	18	
	Structural	10	6	
	Reactive	7	7	
	Undefined	4	3	0.9
Presentation	CS	22	23	
	SE	17	11	0.3
CRI	DZP	25	20	
	PPF	14	14	0.6
History of seizures	No	14	9	
	Yes	25	25	0.4
Previous AED (presence/absence)	No	6	4	
	Yes	19	21	0.5
Previous AED (number)	Monotherapy	11	13	
	Polytherapy	8	8	0.8

**Table 2:** Baseline characteristics of the groups receiving CRI with DZP.

Characteristic		DZP EXP group (*n* = 25)	DZP CTRL group (*n* = 20)	*p* value
Breed	Cross breed	13	5	
	Pure breed	12	15	0.07
Sex	Female intact	2	3	
	Female neutered	9	2	
	Male intact	13	14	
	Male neutered	1	1	0.2
Weight (kg)		22.5 (10–34)	23 (19.5–30.15)	0.6
Age at inclusion (months)		61.5 (31.5–79.25)	54 (37.75–92.25)	0.9
Seizure etiology	Idiopathic	14	12	
	Structural	8	2	
	Reactive	2	3	
	Undefined	1	3	0.2
Presentation	CS	17	17	
	SE	8	3	0.3
History of seizures	No	5	2	
	Yes	20	18	0.4
Previous AED (presence/absence)	No	4	4	
	Yes	16	14	1
Previous AED (number)	Monotherapy	10	10	
	Polytherapy	6	4	0.7

**Table 3:** Baseline characteristics of the groups receiving CRI with PPF.

Characteristic		PPF EXP group (*n* = 14)	PPF CTRL group (*n* = 14)	*p* value
Breed	Cross breed	2	6	
	Pure breed	12	8	0.2
Sex	Female intact	5	4	
	Female neutered	3	2	
	Male intact	5	8	
	Male neutered	1	0	0.7
Weight (kg)		23.25 (12–36)	19.20 (13.38–27.73)	0.5
Age at inclusion (months)		96 (51–116)	49 (21–85)	0.08
Seizure etiology	Idiopathic	4	6	
	Structural	2	4	
	Reactive	5	4	
	Undefined	3	0	0.3
Presentation	CS	5	6	
	SE	9	8	0.7
PPF dosage (mg/kg/min)		0.1 (0.1–0.15)	0.15 (0.1–0.1725)	0.2
History of seizures	No	9	7	
	Yes	5	7	0.4
Previous AED (presence/absence)	No	2	0	
	Yes	3	7	0.2
Previous AED (number)	Monotherapy	1	3	
	Polytherapy	2	4	1

There were no statistically significant differences in clinical outcome between the groups (Table 4).

**Table 4:** Outcomes after CRI with DZP or PPF for 12 h or 24 h.

	CRI duration (12 h vs. 24 h)	ALIVE	DEAD	*p* value
ALL	ALL EXP group (*n* = 39)	23	16	
	ALL CTRL group (*n* = 34)	24	10	0.3
DZP	DZP EXP group (*n* = 25)	17	8	
	DZP CTRL group (*n* = 20)	14	6	0.9
PPF	PPF EXP group (*n* = 14)	6	8	
	PPF CTRL group (*n* = 14)	10	4	0.1

Analysis of length of stay compared the ALL EXP group and the ALL CTRL group. The other subgroups (DZP and PPF) were too small to obtain reliable statistical results. The median length of stay was 56 h (IQR, 40–78) for the ALL EXP group and 58.5 h (IQR, 48–74.5) for the ALL CTRL group. There was no statistically significant difference between the two groups (*p* = 0.8).

Considering the high percentage of dogs affected by IE in both study groups, further statistical analyses were performed on this specific group of patients. Also in this case, no statistically significant difference in clinical outcome were observed (Table 5). The length of hospital stay compared only the ALL IE EXP group and the ALL IE CTRL group due to the small number of subjects in the subgroups. The median length of stay was 45 h (IQR, 36.5–56) for the ALL IE EXP group and 56.5 h (IQR, 48–62.2) for the ALL IE CTRL group. There were no statistically significant differences between the two groups (*p* = 0.1).

**Table 5:** Outcomes after CRI with DZP or PPF for 12 h or 24 h in dogs with IE.

	CRI duration (12 h vs. 24 h)	ALIVE	DEAD	*p* value
ALL IE	ALL IE EXP group (*n* = 18)	13	5	
	ALL IE CTRL group (*n* = 18)	16	2	0.4
DZP IE	DZP IE EXP group (*n* = 14)	11	3	
	DZP IE CTRL group (*n* = 12)	10	2	1
PPF IE	PPF IE EXP group (*n* = 4)	2	2	
	PPF IE CTRL group (*n* = 6)	6	0	0.1

## 4. Discussion

To our best knowledge, this is the first study to evaluate potential associations between different CRI protocols with DZP or PPF and efficacious control of seizure activity or length of hospital stay in a population of dogs presented for CS and SE refractory to baseline emergency treatment.

Exploiting the information obtained from a previous investigation (2), we decided to include in the present study the population of dogs previously described, receiving a standardized baseline treatment protocol and DZP or PPF CRI for 24 h as an historical CTRL group (2) and to compare this group to a prospectively enrolled EXP group of dogs receiving the same baseline treatment protocol and a DZP or PPF CRI for only 12 h.

The decision to set the CRI duration of the EXP group at 12 h was made by combining the available data from the veterinary literature on recommended CRI duration that reports a minimum timeframe between 6 and 12 h (4, 6). Furthermore, a recent study investigating the risk factors for seizure recurrence in a population of hospitalized dogs, revealed that seizures recurred in the first 12 h after hospital admission in up to 90% of cases, regardless of the etiology. This timeframe may provide further justification for the rationale of the present investigation (16). However, due to retrospective nature of the study by Kwiatkowska et al. (16) the seizure treatment protocol was not standardized. As stated by the authors, this aspect may represent an important bias in the correct interpretation of the results and does not allow a direct comparison with our investigation.

The present analysis failed to identify any association between CRI duration (12 h and 24 h) and control of refractory seizure activity nor it could detect any difference in the length of hospital stay between the two groups.

Historically, guidelines for the management of refractory seizure activity in human medicine recommended the induction of therapeutic coma for 24–48 h (7, 17, 18). Nevertheless, recently, debate surrounds whether the induction of therapeutic coma *per se* and its duration may be associated with negative effects such as increased mortality and poor functional outcome, increased risk of complications and prolonged hospital stay (9, 19). Previous studies have produced conflicting results for the efficacy of diverse treatment protocols and the association with mortality, functional outcome and duration of hospital stay (9–14, 20). Several studies conducted on a population of patients referred for SE and receiving different treatment protocol highlighted that the induction of therapeutic coma *per se* was associated with a prolonged hospital stay (9–11), while one recent study concluded that the utilization of therapeutic coma following first-line treatment was associated with reduced SE duration and hospital stay (20). To date however only one retrospective study has evaluated the duration of therapeutic coma as an independent factor for successful treatment of refractory SE and its influence on the duration of hospital stay (14). The duration of therapeutic coma was not associated with a higher risk of poor outcome (either mortality or functional outcome) or complications, whereas protracted therapeutic coma was associated with a higher risk of seizure recurrence following the first weaning attempt. The study concluded that a deeper therapeutic coma of shorter duration could be more effective and safer than the currently recommended duration of 24–48 h (14). No similar conclusions could be drawn from the present study. However, we defined mortality, poor functional outcome, and seizure recurrence as a negative outcome. Even though this aspect represents a limitation to the study, the rationale for this definition was that because electroencephalographic monitoring was not performed, the possible recurrence of non-convulsive seizure activity versus a poor functional outcome due to the seizure activity itself could not be evaluated. It is therefore possible that different results could be obtained with EEG monitoring, for which further studies are warranted.

The length of hospital stay of dogs with favorable outcome did not differ by CRI duration in the present study. Muhlhofer and colleagues reported, however, that length of stay was not influenced by the duration of therapeutic coma *per se* but rather by the drug dosage: patients that received a higher dose of anesthetic had a shorter hospital stay (14). A similar comparison could not be performed because the standardized protocols in the present study differ slightly in dosage, thus precluding confirmation or rejection of this observation in human medicine. Nevertheless, while the length of stay was similar for the two main study groups, we may assume that shorter CRI duration would incur less expense for the medications and overall lower cost for hospital stay.

Previous researches reported a higher fatality rate in case of SE due to structural epilepsy (21, 22). Even though no statistically significant differences were found between groups in terms of etiology, further statistical analyses were performed considering only patients affected by IE to avoid potential bias due to seizure etiology. Also in this case, no significant differences were found in terms of outcome and length of hospital stay. However, the difference in duration of hospitalization between the two groups was consistent, and the lack of statistical results could have been influenced by the relatively low sample size.

In veterinary literature, only another population of patients with refractory seizure activity treated with anesthetic CRI has been reported. In their study, Bray and colleagues reported a median duration of midazolam CRI of 25 h and a median length of hospitalization of 2.5 days (1). When considering only patients receiving 24 h CRI in our investigation, the length of hospital stay was similar between the two studies, but the responder rate was lower compared to that reported by Bray et al. However, the definition of successful outcome differed between the studies, and so did the baseline treatment protocol: our definition of successful treatment was stricter compared to that of Bray and colleagues, and in their study the baseline treatment protocol was not standardized. It is possible that these differences could have accounted for the different results obtained.

Seizure activity is known to progress toward more refractory stages over time (23). One of the mechanisms is the loss of gamma-amino-butyrric acid (GABA)-induced inhibition. In this scenario, GABAergic drug efficacy (e.g., benzodiazepines) may gradually decrease due to reduced synaptic targets (24). In the present study, information on the duration of seizure activity in SE and the number of seizures experienced in CS before treatment was available for the EXP group, but not for a consistent part of the CTRL group. For this reason, we did not include this variable in the analysis. Unfortunately, no conclusions can be drawn from the data collected in the present study.

The demographics of two groups were fairly similar; however, we noted a statistical trend for age at inclusion in the group administered PPF CRI. The EXP group dogs were slightly older than the CTRL group dogs. This difference of age at inclusion in our study population may have partially influenced the outcome for the PPF CRI group. In fact advancing patient age was recently reported as a risk factor for short-term mortality during SE (25).

In the present study, a standardized emergency treatment protocol composed by the association of IV rectal/DZP, IV PB and rectal LEV at a dosage of 40 mg/kg was administered to all patients. This decision was made based on the promising results of a previous open-label clinical trial. In that study, dogs with CS or SE receiving 40 mg/kg of rectal LEV in addition to a standard treatment protocol composed by IV/rectal DZP and IV PB were significantly less likely to develop further epileptic activity compared to those not receiving rectal LEV (26). Since dosages of LEV as high as 60 mg/kg IV have been reported in the literature for the treatment of SE (27) and synergy between LEV and DZP has been shown in both rodent models and human patients (28, 29), we cannot exclude that the utilization of higher dosages of LEV could have positively impacted the outcome of patients requiring a DZP CRI. Further studies are needed to evaluate this hypothesis.

This study has some limitations. No electroencephalographic monitoring of seizure activity was performed, which reduces the power of the study in outcome evaluation. In human medicine EEG is used to define therapeutic strategies and endpoints during SE, even though variability in its interpretation between neurologists have been reported: while some specialists consider the absence of ictal EEG pattern along with the cessation of visible seizure activity as a satisfactory endpoint, others prefer to obtain burst-suppression pattern or complete suppression of EEG background activity (4, 30, 31). In particular, the identification of burst-suppression pattern on EEG has been defined as an indicator of brain inactivation and has been proposed as a tool to guide titration and duration of induced therapeutic coma in human patients with SE. However, the results of a recent study points to the need of developing other strategies for monitoring these patients in light of a very high variability in the amount of EEG suppression achieved, arising from inter and intraspecific individual pharmacokinetic and pharmacodynamic variations (32). In veterinary medicine, only one retrospective study focused on the utilization of EEG monitoring in 7 dogs and 3 cats affected by SE, reporting that EEG seizures continued in all animals after clinical seizures stopped and therefore encouraging the importance of a continuous EEG monitoring for this kind of patients (33). To minimize this shortcoming, we grouped together all clinical conditions potentially associated with a poor outcome, considering as a negative outcome also those patients that did not manifest further clinically evident seizure activity, but did not recover an appropriate state of consciousness after the treatment.

We relied on historical control data for a concurrent control group, which precluded the performance of a proper randomized clinical trial: a population of dogs previously described in a retrospective study by the same authors was in fact used as an historical control group for this prospective study (2). The use of historical control groups has been associated with the risk of baseline differences between groups and selection and outcome assessment bias (34). In the present study, statistical analyses showed that the two groups had similar clinical characteristics, which reduced the potential risk of bias. Also, we used the same criteria for outcome assessment, thus reducing the risk of outcome assessment bias.

In conclusion, even though a shorter DZP or PPF CRI duration was not associated with a worse outcome our study failed to identify a clear superiority of shorter CRI duration on outcome or length of hospital stay. While we recorded no direct effect on outcome and length of hospital stay, shorter CRI duration could have a positive effect on hospitalization costs as concerns quantity of medications administered. Further prospective, randomized clinical trials are warranted to confirm the preliminary results of the present study.

## Data availability statement

The raw data supporting the conclusions of this article will be made available by the authors, without undue reservation.

## Ethics statement

The animal studies were approved by Bioethics Committee of the University of Turin. The studies were conducted in accordance with the local legislation and institutional requirements. Written informed consent was obtained from the owners for the participation of their animals in this study.

## Author contributions

GC and AD’A generated the hypotheses and the experimental design, organized and conducted the study, interpreted and analysed the results, and wrote and revised the manuscript. SF organized and conducted the study, interpreted and analysed the results, and wrote and revised the manuscript. GD and EA conducted the experiment and revised the manuscript. AF interpreted and analysed the results and revised the manuscript. All authors contributed to the article and approved the submitted version.

## Conflict of interest

The authors declare that the research was conducted in the absence of any commercial or financial relationships that could be construed as a potential conflict of interest.

## Publisher’s note

All claims expressed in this article are solely those of the authors and do not necessarily represent those of their affiliated organizations, or those of the publisher, the editors and the reviewers. Any product that may be evaluated in this article, or claim that may be made by its manufacturer, is not guaranteed or endorsed by the publisher.
