# Supplementary material for: Duration of constant rate infusion with diazepam or propofol for canine cluster seizures and status epilepticus
Source: Front Vet Sci. 2023 Aug 22;10:1247100. doi: 10.3389/fvets.2023.1247100 (PMC10478093; doi:10.3389/fvets.2023.1247100)
Supplement: Supplementary file 1 [file Table_1.DOCX]

Supplementary Material

# Supplementary Table 1. Baseline characteristics of the IE study groups

| **Characteristic** |  | **ALL IE EXP group**  **(n=18)** | **ALL IE CTRL group (n=18)** | ***P* VALUE** |
| --- | --- | --- | --- | --- |
| **Breed** | Cross breed | 5 | 6 |  |
|  | Pure breed | 13 | 12 | 0.7 |
| **Sex** | Female intact | 2 | 1 |  |
|  | Female neutered | 6 | 2 |  |
|  | Male intact | 10 | 15 |  |
|  | Male neutered | 0 | 0 | 0.2 |
| **Weight (kg)** |  | 28.7 (19.8-36.9) | 23.7 (22.1 – 33) | 0.7 |
| **Age at inclusion (months)** |  | 55.5 (35.2-75.2) | 41 (18.7-59.7) | 0.07 |
| **Presentation** | CS | 13 | 16 |  |
|  | SE | 5 | 2 | 0.4 |
| **CRI** | DZP | 14 | 12 |  |
|  | PPF | 4 | 6 | 0.5 |
| **History of seizures** | No | 1 | 1 |  |
|  | Yes | 17 | 17 | 1 |
| **Previous AED (presence/absence)** | No | 1 | 1 |  |
|  | Yes | 16 | 16 | 1 |
| **Previous AED (number)** | Monotherapy | 9 | 8 |  |
|  | Polytherapy | 7 | 8 | 0.7 |

**Supplementary Table 2.** Baseline characteristics of the IE groups receiving CRI with DZP

| **Characteristic** |  | **DZP IE EXP group**  **(n=14)** | **DZP IE CTRL group (n=12)** | ***P* VALUE** |
| --- | --- | --- | --- | --- |
| **Breed** | Cross breed | 5 | 3 |  |
|  | Pure breed | 9 | 9 | 0.7 |
| **Sex** | Female intact | 1 | 0 |  |
|  | Female neutered | 4 | 2 |  |
|  | Male intact | 9 | 10 |  |
|  | Male neutered | 0 | 0 | 0.6 |
| **Weight (kg)** |  | 32.7 (21.4-36.9) | 23 (21.9-30.2) | 0.4 |
| **Age at inclusion (months)** |  | 54 (35.2-70.7) | 46 (18-59.2) | 0.2 |
| **Presentation** | CS | 11 | 11 |  |
|  | SE | 3 | 1 | 0.6 |
| **History of seizures** | No | 0 | 0 |  |
|  | Yes | 14 | 12 | NA |
| **Previous AED (presence/absence)** | No | 1 | 1 |  |
|  | Yes | 13 | 11 | 1 |
| **Previous AED (number)** | Monotherapy | 8 | 7 |  |
|  | Polytherapy | 5 | 4 | 1 |

**Supplementary Table 3.** Baseline characteristics of the IE groups receiving CRI with PPF

| **Characteristic** |  | **PPF IE EXP group**  **(n=4)** | **PPF IE CTRL group (n=6)** | ***P* VALUE** |
| --- | --- | --- | --- | --- |
| **Breed** | Cross breed | 0 | 3 |  |
|  | Pure breed | 4 | 3 | 0.2 |
| **Sex** | Female intact | 1 | 1 |  |
|  | Female neutered | 2 | 0 |  |
|  | Male intact | 1 | 5 |  |
|  | Male neutered | 0 | 0 | 0.1 |
| **Weight (kg)** |  | 21.5 (17-31.4) | 28.7 (22.6-34.5) | 0.4 |
| **Age at inclusion (months)** |  | 67.5 (43.7-90.7) | 37 (24.7-54.5) | 0.2 |
| **Presentation** | CS | 2 | 5 |  |
|  | SE | 2 | 1 | 0.5 |
| **History of seizures** | No | 1 | 1 |  |
|  | Yes | 3 | 5 | 1 |
| **Previous AED (presence/absence)** | No | 0 | 0 |  |
|  | Yes | 3 | 5 | NA |
| **Previous AED (number)** | Monotherapy | 1 | 1 |  |
|  | Polytherapy | 2 | 4 | 1 |
